# Development and Evaluation of a Hallucination Awareness Scale for Healthcare Professionals and its impact on diagnostic confidence

**DOI:** 10.3389/fdgth.2026.1772345

**Published:** 2026-03-17

**Authors:** Urvashi Tandon

**Affiliations:** Chitkara Business School, Chitkara University, Punjab, India

**Keywords:** extrinsic hallucinations, generative AI, hallucinations, healthcare, intrinsic hallucinations, diagnostic confidence

## Abstract

Generative artificial intelligence (Gen AI) has gained immense significance in recent years, particularly in the field of healthcare. Despite its significant role in streamlining healthcare-related tasks, there remain unanswered concerns regarding the challenges of incorporating this technology into healthcare settings and it effect on diagnostic confidence. The purpose of this research is to address this gap by developing and validating a comprehensive scale that captures risks like hallucinations and measures their impact on diagnostic confidence among healthcare practitioners. A three-step process was carried out to develop the scale. Data were collected from healthcare professionals and analyzed using exploratory factor analysis and confirmatory factor analysis. In the third step, structural equation modeling using SmartPLS was applied to validate the hypothesized relationships. The results indicated a significant impact of awareness of extrinsic hallucinations on diagnostic confidence. However, awareness of intrinsic hallucinations showed no significant impact on diagnostic confidence. This research contributes to the existing literature by examining the risks associated with Gen AI by validating and developing a reliable scale to measure the challenges healthcare practitioners face when using Gen AI tools.

## 1 Introduction

A radical transformation is underway as large language models (LLMs) are penetrating the healthcare sector, creating new opportunities in the field (1, 48). These foundation models have opened new avenues for decision support, particularly in relation to health system operations. However, their integration into the complex healthcare system has led to the emergence of critical challenges for the sector, thereby influencing diagnostic decision-making. A common and specific concern is hallucinated output generated by LLMs, which seems reasonable but is factually incorrect or contrived (2, 50). This inappropriate, incongruous, and fabricated information may pose risks, such as incorrect diagnoses and inappropriate medication, which may immediately alter diagnostic decision-making (3, 4). As practitioners operate in highly complex environments with high information density, time pressure, and dynamic technological interfaces, these environments at times result in domain-specific imprecision and inaccuracies that mislead diagnosis and clinical judgment. For example, LLMs may alter patient symptoms, history, or clinical findings, thereby producing a clinical note that does not exist in the medical records. A physician may selectively respond to information that supports an initial diagnostic impression, which may distort clinical judgment, resulting in an inappropriate diagnosis and treatment.

**Table 1:** Participant characteristics and themes generated through qualitative interviews.

Participant ID	Gender	Professional role	Years of professional experience	Quotes	Themes
R1	Female	Physician	10	A patient visited me last month where he consulted AI for mild headache she was having while the output indicated “possible brain tumor”. Patient panicked after seeing the AI generated output; however with my intervention she was satisfied. This exaggerated output of AI created anxiety among patients	Extrinsic hallucinations
R2	Male	Physician	12	I began to notice that the AI would sometimes take a few familiar symptoms and quickly stretch them into a broad conclusion, even when the case had important differences. It felt like the system was relying on a template rather than the full context. Seeing this happen repeatedly made me aware that the model tends to overgeneralize patterns it has learned, especially common or high-frequency ones. That awareness changed how I interact with its suggestions I'm more cautious when a recommendation feels too familiar or too neatly packaged. Instead of assuming the pattern fits, I actively look for what doesn't align. Recognizing this kind of intrinsic hallucination helped me understand the limits of pattern-based reasoning in AI, even when the output sounds confident and well-structured	Pattern overgeneralizations
R3	Female	Oncologist	7	I recently came across with a AI-generated radiology report where benign calcification was labeled as “early stage carcinoma”. This report was incorrect and human intervention was required to draw the actual inference from the report. Relying on such reports may lead to potential harm via miscommunication. This indicated diagnostic exaggerated content leading to textual hallucinations	Extrinsic hallucinations
R4	Male	Orthopedic surgeon	6	As an orthopedic surgeon, I reviewed an AI-assisted clinical decision support output. In one instance, the system generated the treatment recommendation for a middle-aged patient with mild knee pain as “advanced osteoarthritis with knee replacement.” On clinical examination, the patient could be treated with conservative management rather than surgical treatment	Biased and faulty output
R5	Female	Gynecologist	9	AI misinterpreted high vitamin B12 as deficiency, and suggested supplementary diet to enhance Vitamin B12 in my patient. However, I had to correct the report while prescribing medicines to the patient. This is infact, misinterpretation of lab data leading to language fluency masking factual inaccuracy. While the prompt was clear (please interpret the report), AI failed to diagnose it properly, leading to inaccurate output and confusion	Intrinsic hallucinations
R6	Male	Physician	11	What really caught my attention was how the AI sometimes made a situation seem more clear-cut than it actually was. The way it framed the information subtly pushed certain details into focus while downplaying others, even though all the data was technically correct. Over time, I realized this was an intrinsic issue the model's internal reasoning was shaping a distorted “picture” of the case. That experience made me more aware of how AI outputs can influence perception, not by inventing data, but by emphasizing patterns in a way that alters how a clinical situation is interpreted. It reminded me that even well-structured recommendations can create perceptual distortions, and that I need to consciously step back and reassess the full clinical context rather than letting the AI's framing guide my interpretation	Perceptual distortion
R7	Male	Nephrologist	12	Becoming aware of intrinsic hallucinations actually had the opposite effect on my diagnostic confidence. When I realized that the AI could generate internally coherent but clinically unfounded conclusions—even when the input data was correct—it made me more cautious rather than confident. Unlike documentation or data issues, intrinsic hallucinations are harder to trace back to a specific source or fix in real time. When an AI sounds convincing but I can't clearly explain why it arrived at a particular conclusion, it introduces uncertainty into the decision-making process. That awareness didn't help me feel more confident in the diagnosis; instead, it reinforced the need to rely more heavily on my own clinical judgment and independent verification, which slowed decision-making and reduced confidence in using the AI output as part of the diagnostic process	Diagnostic confidence
R8	Male	Physician	6	While using an AI system to assist with a situation in an emergency care setting, I encountered a case where the model suggested acute renal failure based on elevated creatinine levels. Because I was aware of how hallucinations can occur particularly when AI systems misinterpret temporally misaligned or incomplete data, I reviewed the source of the laboratory values instead of accepting the recommendation at face value. I discovered that the creatinine result belonged to a previous admission and had been incorrectly associated with the current encounter. Once this hallucination was identified and corrected, I was able to confidently rule out acute renal failure and proceed with an alternative diagnosis supported by the patient's current clinical presentation. This experience reinforced that awareness of AI hallucinations enhances diagnostic confidence by enabling clinicians to verify data, understand the limits of the system, and make decisions grounded in validated information rather than uncertainty or blind trust	Diagnostic confidence
R9	Male	Physician	10	There was a point when I noticed the AI flagging high risk in patients who, clinically, just didn't look unwell. At first, it made me second-guess myself, but instead of assuming the system was overreacting, I started digging into where the information was coming from. I realized that some of the vitals and lab values it was using were delayed or pulled from older notes that hadn't been updated yet. That was a turning point for me. It made me realize that the issue wasn't the model's logic, but the quality and timing of the data feeding into it. Since then, I've become much more conscious of checking timestamps, data sources, and documentation gaps before trusting any AI output. That experience really shaped my awareness of how easily data quality issues can translate into misleading AI recommendations	Awareness of data quality risks
R10	Male	Physician	11	Over time, I started noticing that some AI recommendations were consistently skewed in certain directions, even when the patient data looked complete and accurate. That made me realize these weren't documentation or data issues, but something internal to how the model was reasoning. Seeing the AI confidently suggest patterns that didn't fully align with clinical reality made me more aware that the system carries its own biases—shaped by training data, assumptions, and dominant patterns it has learned. That awareness changed how I interpret AI outputs. I no longer see them as neutral or purely objective; instead, I consciously ask myself whether a recommendation might be influenced by underlying biases in the model's training or logic. In that sense, intrinsic hallucinations helped me become more alert to bias, even if they didn't increase my confidence in the diagnosis itself	Awareness of biases
R11	Male	Physician	8	I remember a situation where the AI kept highlighting a condition that didn't quite match what I was seeing with the patient. Instead of assuming the system was wrong, I went back to the notes it was using and realized some of the documentation had been copied forward from a previous visit and never corrected. The AI treated that outdated information as current, which led to a misleading suggestion. That experience made me much more aware of how small documentation errors—like copy-paste habits or updated problem lists—can directly affect AI outputs. Since then, I pay closer attention to the accuracy of clinical notes, knowing that even minor documentation issues can snowball into significant AI misinterpretations	Awareness of documentation errors
R12	Male	Physician	9	I am just recalling a moment when AI tool suggested that the symptoms of patient were psychosomatic due to anxiety, notwithstanding the absence of any documented psychiatric history and screening results. This biased output risked prematurely tapering the diagnostic pathway and delayed investigations	Awareness of biases

**Table 2:** Demographic profile.

Demographic characteristic (*n* = 452)	Frequency	Percentage
Gender		
Male	298	65.93
Female	154	34.07
Educational qualification		
MBBS	196	43.36
MD	207	45.80
Others	49	10.84
Age (years)		
25–29	179	39.60
30–45	232	51.33
Above 45	41	9.07
How long have you been using Gen AI		
Less than 1 year	252	55.75
1–2 years	186	41.15
More than 2 years	14	3.10
Purpose of using Gen AI		
Interpreting reports	115	25.44
Personalizing treatment plans	105	23.23
Accelerating drug discovery	153	33.85
Administration tasks	79	17.48

Domain-specific inaccuracies or hallucinations in the medical field represent a novel area that remains underexplored in the existing literature. Hallucinations, which encompass a broader spectrum of distortions, may originate from both external and internal sources (5). Intrinsic hallucinations emerge from internal cognitive tendencies, such as biases and patterns, which may lead practitioners to rely on misleading cues (6). In contrast, extrinsic hallucinations originate from external information sources, including poor data quality or system-level inconsistencies in clinical records (6). If practitioners are unaware of their influence, both forms of hallucinations can destabilize and compromise the reliability of diagnostic judgments. Despite the immense significance of awareness of hallucinations, to the best of the author's knowledge, no validated instrument currently exists to systematically measure sensitivity and awareness of practitioners to both extrinsic and intrinsic hallucinations, as well as their impact on diagnostic confidence. Very few researchers have developed and validated scales related to Gen AI. For example, Hoffmann et al. (7) validated a scale measuring perceived value, Ivanov et al. (8), developed and validated a scale assessing attitudes toward ChatGPT, and Gao et al. (9) integrated self-determination theory with the interactive–constructive–active–passive framework to study the behavior of Chinese students. Although existing studies have meticulously validated attitudes and cognitive behaviors toward Gen AI, a validated scale that captures challenges and risks widely discussed in the literature has yet to be developed in this context. Several notable scholarly readings have studied Gen AI across sectors such as knowledge management (10), tourism (11), and medicine (5). However, most of these studies remained focused on opinions, concepts, or review articles (51). Furthermore, the majority of studies emphasize adoption-related aspects, with limited consideration of the quality of the output generated by Gen AI (1).

To address this gap, the present study draws on the perspectives of healthcare practitioners and the existing literature on hallucinations to develop and validate a Hallucination Awareness Scale (HAS) for Healthcare Professionals. This scale is designed to capture multiple dimensions of hallucination awareness, including awareness of biases, pattern overgeneralization, data quality risks, and overgeneralization of the output received.

The study offers numerous implications for healthcare professionals. First, hallucination is conceptualized as a higher-order construct covering both intrinsic and extrinsic hallucinations within the healthcare context. The scale developed in this research is statistically validated and demonstrates how awareness of intrinsic and extrinsic hallucinations can enhance diagnostic confidence, contributing to safer, more appropriate diagnoses. The insights derived from the development of the HAS may provide a roadmap for healthcare professionals, guiding them in the use of Gen AI during patient consultations. Therefore, this research addresses the research gap in the Gen AI healthcare literature and provides insights for healthcare professionals on integrating Gen AI into routine diagnostic practices while exercising utmost care, with the theory of epistemic risk as a theoretical underpinning.

## 2 Theoretical underpinning

A comprehensive literature review was performed in several stages. To understand the concept of hallucinations, the theory of epistemic risk was considered as the theoretical foundation for the first stage. To comprehend the major patterns and how they have developed across the academic literature, pertinent literature on various issues is discussed.

### 2.1 Epistemic risk theory

Risk is commonly understood as “any unsolicited event that may compromise accuracy and precision” (52). The increasing use of Gen AI in the healthcare sector has produced significant outputs and enabled diagnostic assistance but has also introduced significant risks and concerns related to inaccurate hallucinated information, diagnostic misclassification, and incorrect treatment recommendations (53).

The theory of epistemic risk focuses on the risks or obscurities associated with accuracy, precision, and knowledge (54). Epistemic risks are shaped by an individual’s comprehension and convictions and, hence, differ from conventional forms of risk. Epistemic risk theory (54, 55) provides a robust conceptual foundation for examining how healthcare professionals may be exposed to unreliable, distorted, or misleading AI-generated outputs. This theory is particularly significant for understanding the non-deterministic nature of Gen AI outputs, which can increase the likelihood of unreliable, distorted, or misleading information being presented as credible. Due to fallacious reasoning, psychological prejudices, inadequate evidence, limitations in understanding, or skeptical views regarding the reliability of the findings, such claims may lead to inappropriate diagnoses and treatment decisions. Previous researchers have successfully applied epistemic risk theory to examine risks related to Gen AI (6, 56), further supporting its validity as a guiding framework in this context.

This research explains hallucination as perceived risks associated with the use of Gen AI, drawing on the theory of epistemic risk. The theory emphasizes the potential threats to information procurement when sources are untrustworthy, deceptive, or inaccurate (55). When healthcare professionals use Gen AI for diagnostic purposes, the generation of fabricated or factually incorrect information directly introduces epistemic risk by compromising diagnostic precision. For instance, if healthcare professionals integrate hallucinated outputs into their diagnostic decisions, such decisions may be jeopardized. This aligns with the premise of the epistemic risk theory of flawed epistemic environments. Such conditions threaten epistemic reliability and justification, which are core concerns of the theory of epistemic risk. Awareness of hallucinations enables practitioners to thoroughly evaluate AI outputs rather than accepting them blindly. Healthcare professionals who can distinguish between fabricated and misleading information can maintain epistemic control over diagnostic procedures, leading to enhanced diagnostic confidence.

## 3 Methods

### 3.1 Phase 1

A qualitative study was conducted to understand the dimensions of hallucinations. To develop respective scale items, semi-structured interviews were conducted with the healthcare practitioners who use Gen AI in their day-to-day decision-making and diagnosing patients. These interviews helped identify challenges faced by healthcare professionals when using Gen AI, which were subsequently validated through a quantitative study.

#### 3.1.1 Interview design

Data were collected using one-to-one, in-depth, semi-structured interviews. Respondents were allowed to share their perspectives without any hesitation and were assured of the academic nature of the study. The interview questions were carefully designed to extract the desired information regarding hallucinations associated with AI. The introductory part briefed participants on the theme of the study and assured them of the anonymity of their responses. Participants were also assured that their responses would be used strictly for academic purposes. The second part covered questions about their experiences in identifying hallucinated outputs while using Gen AI in patient treatment. Their responses were recorded after obtaining their consent. The interviewing procedure was concluded after 12 interviews due to a lack of new insights (12). This decision was based on the principles of information power and thematic saturation rather than statistical representativeness. For example, later interviews restated similar experiences, such as inclined outputs, inappropriate patterns, and distorted information, without contributing new interpretive categories. Following the completion of all interviews, the responses were paraphrased to elucidate the inputs provided by the respondents. Hence, this sample was considered appropriate to support the exploratory objectives of Phase 1 of the study.

In line with COREQ Domain 1 (Research team and Reflexivity), all phases of this research, comprising qualitative and quantitative analyses, were conducted by a single author, ensuring consistent reflexive engagement throughout the research process. The author was exclusively responsible for designing the interview protocol, conducting and transcribing the interviews, and performing the qualitative analysis. The researcher is a female academic holding a doctorate degree and has prior experience in conducting mixed-methods research. She is currently employed as an academician at a university. No prior association existed between the interviewer and interview participants. Interactions between both the interviewer and interviewees were strictly limited to research-related communication. Participants were clearly informed about the academic nature of the study and its research objectives. This lack of a pre-existing relationship helped overcome social desirability bias. Notes were prepared throughout the interview and analysis phases to support data interpretation and identify potential influencing factors.

#### 3.1.2 Study sample

Snowball sampling was employed to reach potential healthcare professionals who use Gen AI to treat their patients. The sample comprised eight men and four women. Snowball sampling was preferred in qualitative research due to the evolving nature of the phenomenon under investigation.

#### 3.1.3 Data analysis and qualitative analysis approach

The qualitative analysis was conducted by a single author, who was entirely responsible for coding, theme development, and data interpretation. No additional researchers were involved in this analytic process.

The transcripts were not returned to the participants to minimize participant burden. The focus of this research was on identifying conceptual patterns that authentically support individual-level precision. Hence, returning transcripts was avoided to reduce participant burden.

Formal triangulation involving multiple analysts or data sources was not implemented, as this phase was designed as an exploratory phase with a single researcher performing qualitative and quantitative analyses. However, academic rigor was ensured through engagement with the data and repeated comparisons with the emerging themes. Participants were not involved in validating the final interpretations. An important limitation of qualitative research is its subjective nature (13, 14). Hence, to mitigate the risk of social judgment and to avoid altering the original responses by the participants, final interpretations were not sent to them. Further, reviewing the interpretations may be time-consuming, and participants may have limited patience for theory-driven analyses. Hence, rigor was ensured through sustained reflexive engagement with the data and transparent documentation.

The next step involved identifying relevant themes related to hallucinations. Thematic analysis was employed for this purpose due to its ability to describe, analyze, and report that data (15). To derive pertinent themes, three steps were taken: transcription, coding, and data processing. By summarizing the answers provided by the respondents, themes were identified, and the literature was searched for coverage of these themes (Table 1). A methodical process was followed for thematic analysis (16). The first phase involved creating and identifying the codes; this was followed by compilation of codes, with efforts made to generate a common theme among them. The themes were rechecked for alignment with the existing literature.

##### 3.1.3.1 Content validity

A three-step approach was followed to establish the content validity of the themes that emerged from the literature review. First, the interview guide was prepared by reviewing the extant literature. This interview guide was reviewed by healthcare professionals and academicians to ensure adequate coverage and clarity of the experiences of healthcare professionals with Gen AI. Data were collected through probing to extract relevant themes. Second, thematic analysis was conducted to identify patterns that emerged from rigorous evaluation across the interviews, and it was ascertained that the emerging themes were distinct and represented the constructs. These themes were subsequently translated into scale items, with due consideration given to simplicity and association with the conceptual definition of the constructs. Where possible, item wording reflected expressions of the participants to preserve contextual authenticity. This qualitative expert interaction process established conceptual representativeness. To assess the viability of the study, these findings were discussed with health practitioners and a few AI specialists. These notes were referred to throughout the study to reflect on the decisions regarding the relevance of the construct.

### 3.2 Phase 2

#### 3.2.1 Instrument design

On the basis of inputs received from the interviews and literature, hallucinations were considered as a higher-order construct. The scale items were framed by incorporating expert opinions gathered during the interviews and evidence from the existing literature. Further, the study by Ng and Palmer (17) was referenced for developing the scale items related to diagnostic confidence. Utmost care was taken to ensure that the scale items covered all themes and viewpoints that emerged from the qualitative study. After framing the scale items, a thorough check was conducted to confirm that all codes and themes are covered. This scale was further discussed with healthcare professionals and AI experts for content and face validity. These experts evaluated each scale item for clarity. A few double-barreled scale items were reframed. A few items that did not reflect the construct domain were eliminated. All language-related issues were carefully addressed, and suggestions from this group were incorporated to improve the clarity of the developed scale (Supplementary Table 1). A pilot study was also conducted with 35 healthcare professionals to assess the face and content validity of the scale. Reliability was evaluated using Cronbach's alpha for each construct, and all values exceeded the recommended threshold of 0.7. These validations were followed by actual data collection.

#### 3.2.2 Sampling strategy and data collection

Since the sampling frame was unknown in this context, non-probability sampling techniques were preferred to locate respondents. Purposive sampling was preferred, as it assists the researchers in identifying respondents with adequate information about the domain. Sample size was determined by using the threshold proposed by Kline (18), who suggested an N:Q ratio of 20:1. Therefore, a sample size of 452 was considered for this research. The target population was healthcare professionals who are actively using AI for patient diagnosis and clinical decision-making. Data were collected through multiple sources. An online Google Form was created and circulated among healthcare professionals. In addition, personal visits were made to certain clinics and hospitals. Practitioners were also approached during healthcare-related conferences. Participation was voluntary, and respondents were assured of the anonymity of their responses. Ethical guidelines were followed throughout the study, and participants were allowed to withdraw at any point in time.

#### 3.2.3 Demographic profile

The sample demonstrated gender representation (males: 65.93% and females: 34.07%). The majority of respondents held an MD qualification (45.80%), were between 30 and 45 years of age, and had been using Gen AI for 1 year (Table 2).

## 4 Refining research hypotheses and explaining the conceptual framework

This section elaborates on how the themes that emerged from the thematic analysis facilitated the development of hypotheses. Thematic analysis assisted in understanding the experiences of healthcare professionals and in providing real-time episodes that theories of technology adoption may not explain, given the evolving and dynamic nature of AI.

### 4.1 Intrinsic hallucinations and diagnostic confidence

Hallucinations appear due to the irrational, technically inaccurate output produced by large language models like ChatGPT (19). Kim et al. (1) further mentioned medical hallucinations as “any model generated output that is factually incorrect, logically inconsistent, or unsupported by authoritative clinical evidence in ways that could alter clinical decisions.” Hallucinations in healthcare are more closely related to diagnostic decisions, therapeutic advice, or the forecasting or elucidation of laboratory findings, where errors have an immediate impact on patients (20, 21). The study by Asgarie et al. (2025) further emphasized the domain-specific nature of hallucinations in the healthcare sector, which can be challenging to recognize without expertise. Reliance on AI might lead to errors and potentially delay decision-making, as highlighted by Ly et al. (22).

Researchers have further emphasized that the impact of hallucinations in the medical field can be severe, as errors may lead to ambiguous diagnoses and inappropriate treatment decisions, which in turn harm patients (21, 23, 24). Hence, expert intervention is needed to detect hallucinated outputs generated by LLMs, as indicated by a previous study by Xu et al. (57). Medical hallucinations not only compromise the health of patients but also undermine confidence in AI-assisted clinical systems.

Previous studies have emphasized the problems caused by misinformation and faulty outputs from GEN AI (4, 25). The study by Tandon et al. (24) further confirmed that biases due to Gen AI shape the diagnostic decisions of healthcare professionals. When healthcare practitioners develop awareness and the skills necessary to identify and address algorithmic biases, they are less likely to rely on shortcuts that may misinterpret the diagnosis.

Apart from this, healthcare professionals also perceive patterns that are imperfect, unpredicted, and clinically irrelevant. This leads to overdiagnosis or incorrect diagnosis, which could be fatal for the patients. However, awareness of such overgeneralizations enables healthcare professionals to verify and validate patterns based on their experiences (26).

Similarly, healthcare professionals can recognize distortions and evaluate their interpretations. Hence, awareness of biases, patterns, and perceptual distortions enables practitioners to monitor intrinsic hallucinations (27, 28). Such awareness of intrinsic hallucinations strengthens the confidence of healthcare practitioners in treating patients.

H1: Awareness of biases, pattern overgeneralizations, and perceptual distortions are indicators of intrinsic hallucination awareness.
H2: Intrinsic hallucination awareness has a significant positive effect on diagnostic confidence.

### 4.2 Extrinsic hallucinations and diagnostic confidence

Extrinsic hallucinations, on the other hand, arise from inaccuracies in the external sources. These inaccuracies may emerge from missing data, transcription errors, and low-quality inputs (29, 30). Awareness among healthcare professionals to identify errors in data contributes to increased diagnostic confidence. When healthcare practitioners are aware of data quality risks caused by incomplete information, outdated records, and system-generated errors, this awareness encourages them to evaluate the reliability and correctness of data rather than treating it as accurate. Practitioners who are aware of data quality risks are better prepared to detect extrinsic hallucinations, thereby enhancing their diagnostic confidence.

Similarly, omissions and duplication of data may lead to documentation errors (31, 32). Such errors can also arise from incorrect entries, requiring practitioners to remain attentive. An observant and aware attitude prompts practitioners to cross-check information, increasing their ability to identify extrinsic hallucinations embedded within external data sources. Based on the above discussion, the following hypotheses are posited:

H3: Awareness of data quality risks and awareness of documentation errors are indicators of extrinsic hallucination awareness.
H4: Extrinsic hallucination awareness has a significant positive effect on diagnostic confidence.

## 5 Results

### 5.1 Phase 1

The results of qualitative analysis indicated two types of hallucinations (intrinsic and extrinsic). Hallucinations refer to irrational or inappropriate content generated by Gen AI. Such hallucinations may develop due to model architecture and training purposes, as suggested by Alkaissi and McFarlane (33). Ji et al. (2) classified hallucinations into two types: intrinsic (where the output of LLMs contradicts the original content) and extrinsic (where the generated content cannot be validated against the original material). Hallucinations may arise from training data, as suggested by previous researchers (34, 35). Intrinsic hallucinations may be detected by evaluating the relationship between generated and source content, while assessing the created content against external information sources is a simple way to identify extrinsic hallucinations (36).

### 5.2 Phase 2

#### 5.2.1 Initial data quality checks

Before conducting actual data analysis, initial data quality checks were performed. The entire dataset was checked for missing values. Missing values were found to be less than 1%. For the distributional assumption, we checked the skewness and kurtosis values of the indicators, which fall well within the recommended threshold range of +3 to −3, indicating a normal distribution of the data and the adequacy of the data for parametric analyses (37) (Supplementary Table 2). The mean of all scale items was above 3.5, indicating that the users recommended the scale items. Common method bias was assessed using Harman’s single-factor test, which accounted for 39.74% of the total variance across all the variables. Since the value is below the suggested threshold, common method bias was not considered a significant concern in this research. CMB was further examined using the variation inflation factor. As a rule of thumb, a VIF value greater than 5 indicates potential multicollinearity issues. In this study, all VIF values were below this threshold, suggesting that multicollinearity was not a severe issue and that the data were adequate for further analysis (Table 3).

**Table 3:** Exploratory factor analysis.

KMO and Bartlett's test of sphericity		
Kaiser–Meyer–Olkin measure of sampling adequacy	0.899	
Bartlett's test of sphericity	Approx. chi-square	3,630.233
	Df	276
	Sig.	0.000

Items	1	2	3	4	5	6	Cronbach's alpha
I3	0.830						0.845
I2	0.823						
I1	0.813						
I4	0.770						
I6		0.739					0.815
I5		0.725					
I7		0.713					
I8			0.808				0.798
I10			0.799				
I9			0.788				
I11			0.736				
I13				0.835			0.823
I14				0.828			
I12				0.814			
I18					0.817		0.814
I16					0.770		
I15					0.745		
I17					0.556		
I22						0.832	0.768
I23						0.696	
I21						0.597	

Extraction method: principal component analysis.

Rotation method: Varimax with Kaiser normalization.

Rotation converged in 10 iterations.

Total variance explained: 74.529.

#### 5.2.2 Structural validity of initial scale items

Initial scale items were subjected to content and face validity. Exploratory factor analysis (EFA) was performed using principal component analysis (PCA), which was followed by confirmatory factor analysis (CFA). EFA and CFA were performed on different sample sets, as suggested by previous studies (38).

Due to the multidimensional nature of the constructs validated in this research and the sample size of 452, we preferred a random split-sample methodology to leverage the strengths of both exploratory and confirmatory factor analyses. Random splitting was employed to preserve population representativeness and maintain comparable covariance, allowing CFA to function as a true validation of EFA (39). The split was conducted using a randomized procedure in IBM SPSS version 27, wherein a random number was generated for each respondent and cases were assigned to two independent groups. This enabled the probability of being apportioned to either subsample to be equivalent. Approximately half of the cases were considered for EFA, and the remaining were used for CFA. For exploratory analysis, scale items with a factor loading of 0.5 were retained (40). Items with a value less than the threshold of 0.5 were removed. After performing EFA, internal consistency of the scale items was measured using Cronbach's alpha, and the constructs showed values greater than 0.7, validating the reliability of the scale items (41) (Table 3). Item-wise descriptive statistics were compared using independent-sample *t*-tests. No statistically significant differences were observed, indicating that both samples are distinct.

Exploratory factor analysis was performed using PCA. Prior to factor extraction, sampling adequacy was assessed using the Kaiser–Meyer–Olkin measure and Bartlett's test of sphericity. Factors with eigenvalues greater than 1 were retained and subjected to confirmatory factor analysis (42). Given the conceptual relatedness of the latent constructs, varimax (orthogonal) rotation was preferred due to the uncorrelated nature of the constructs.

The remaining data set was subjected to CFA using SmartPLS. Partial least-squares structural equation modeling (PLS-SEM) was preferred in this research due to the exploratory nature of the study. The primary purpose of PLS-SEM is to predict the key driver constructs or indicators through component expansion (43, 58). Since the data were collected using a Likert scale, SmartPLS was used for analysis. The PLS-SEM algorithm treats Likert-type indicators as approximately continuous, employs a linear variance-based approach, and allows Pearson correlations among items.

Since the objective of this research was to develop a hallucination scale and to explore its impact on diagnostic confidence, PLS-SEM was preferred over CB-SEM, which is used for theory testing or confirmation of existing theories. While conducting a confirmatory factor analysis, items with a standardized outer loading threshold of 0.6 were considered (18). However, none of the items reported outer loadings below 0.6; hence, all the items were retained for further analysis. Similarly, the average variance extracted ranged between 0.654 and 0.803; both composite reliability and Cronbach's alpha values exceeded the recommended threshold of 0.7 for all constructs (Table 4). Furthermore, discriminant validity was established, as the square root of the AVE was greater than the constructs (Table 5).

**Table 4:** Confirmatory factor analysis.

Construct	Indicator	Outer loading	Cronbach's alpha	Composite reliability (rho_a)	Composite reliability (rho_c)	Average variance extracted (AVE)
Awareness of data quality risks	I1	0.792	0.830	0.835	0.887	0.662
	I2	0.825				
	I3	0.857				
	I4	0.779				
Awareness of documentation errors	I5	0.843	0.876	0.883	0.924	0.803
	I6	0.932				
	I7	0.784				
Awareness of biases	I8	0.902	0.844	0.866	0.898	0.692
	I9	0.883				
	I10	0.889				
	I11	0.622				
Awareness of pattern overgeneralizations	I12	0.747	0.784	0.804	0.874	0.699
	I13	0.886				
	I14	0.802				
Perceptual distortion	I15	0.818	0.737	0.744	0.850	0.654
	I16	0.824				
	I17	0.784				
	I18	0.902				
Diagnostic confidence	I19	0.889	0.833	0.837	0.900	0.749
	I20	0.859				
	I21	0.849				

**Table 5:** Discriminant validity (Fornell–Larcker criteria).

Variables	Diagnostic confidence	Awareness of data quality risks	Awareness of documentation errors	Awareness of biases	Pattern overgeneralizations	Perceptual distortions
Diagnostic confidence	**0** **.** **866**					
Awareness of data quality risks	0.587	**0** **.** **814**				
Awareness of documentation errors	0.030	0.235	**0** **.** **896**			
Awareness of biases	0.058	0.348	0.712	**0** **.** **832**		
Awareness of pattern overgeneralizations	0.118	0.369	0.647	0.705	**0** **.** **836**	
Perceptual distortions	0.365	0.391	0.367	0.419	0.497	**0** **.** **809**

Items in bold represent square root of AVE.

**Figure 1:**
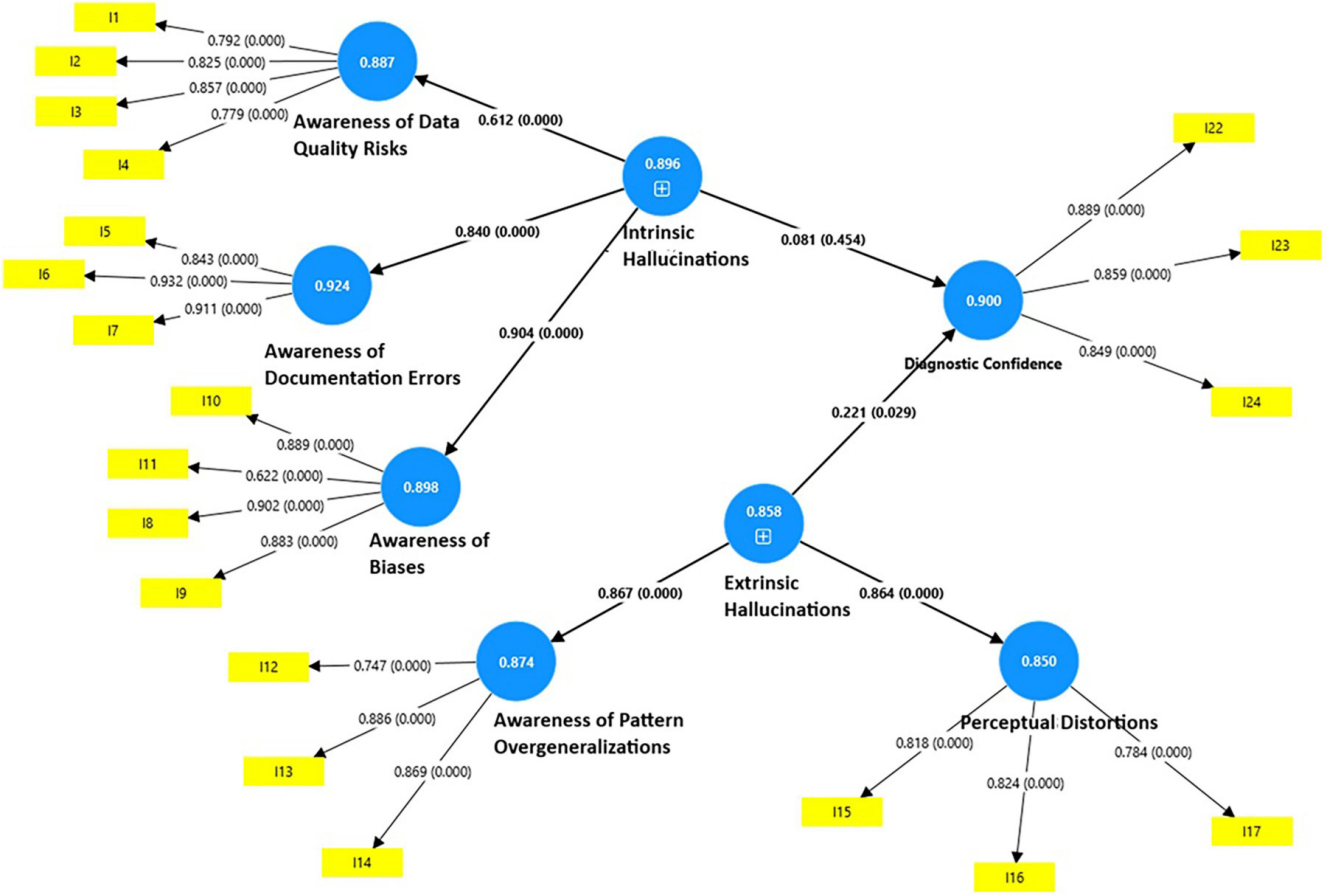
Comprehensive model.

Table 6 and Figure 1 present the path relationships among the endogenous constructs. Extrinsic hallucinations were associated with a significant positive impact on diagnostic confidence (*β* = 0.221, *t* = 2.181, *p* = 0.029), suggesting that greater awareness of extrinsic hallucinations enhances healthcare practitioners’ diagnostic confidence and supports accurate decision-making. Further, both sub-constructs of extrinsic hallucinations, namely, awareness of data quality and awareness of documentation errors, predict extrinsic hallucination awareness, as evidenced by their heavy loadings on the main construct of extrinsic hallucinations.

**Table 6:** Path relationships.

Hypothesized relationships	Path coefficients	Standard deviation (STDEV)	T statistics (|O/STDEV|)	*P* values	*F*-square	Result
Extrinsic hallucination awareness→ diagnostic confidence	0.221	0.101	2.181	0.029	0.151	Supported
Extrinsic hallucination → awareness of data quality risks	0.867	0.020	43.661	0.000	3.016	Supported
Extrinsic hallucination → awareness of documentation errors	0.864	0.015	57.632	0.000	2.933	Supported
Intrinsic hallucination awareness → diagnostic confidence	0.081	0.108	0.748	0.454	0.003	Not-supported
Intrinsic hallucination → awareness of biases	0.612	0.059	10.357	0.000	0.599	Supported
Intrinsic hallucination → awareness of pattern overgeneralizations	0.840	0.024	35.190	0.000	2.404	Supported
Intrinsic hallucination → perceptual distortions	0.904	0.017	54.353	0.000	4.488	Supported

Surprisingly, in contrast, intrinsic hallucinations did not have a significant impact on diagnostic confidence (*β* = 0.081, *t* = 0.748, *p* = 0.454), suggesting that awareness of internally driven cognitive distortions alone may not directly translate into increased confidence in diagnostic judgments. All three sub-constructs loaded significantly on the higher-order construct of extrinsic hallucination. Among these, perceptual distortions demonstrated the highest loading (*β* = 0.904), followed by awareness of pattern overgeneralizations (*β* = 0.840). Surprisingly, although awareness of biases (*β* = 0.612) also loaded on the higher-order construct, its loading was comparatively lower than the other two sub-constructs. These results underscore the complementary yet distinct roles of extrinsic and intrinsic hallucination awareness in enhancing diagnostic confidence. The model fit indices indicated an adequate fit, with the standardized root-mean-square residual value of 0.09 and an NFI of 0.91. The *F*-square effect varied from negligible to very large, reflecting varying levels of substantial explanatory power among the predictors. Intrinsic hallucinations demonstrated a negligible effect, while extrinsic hallucinations exhibited a medium effect.

## 6 Discussion and conclusions

This study develops and validates a scale of challenges associated with the use of Gen AI by surveying a sample of healthcare professionals. The study further validates the impact of awareness of extrinsic and intrinsic hallucinations on diagnostic confidence. To the best of the author's knowledge, this is one of the initial studies to develop a scale of hallucinations and to further validate their impact on diagnostic confidence. Hallucinations were validated as a multidimensional construct, with intrinsic and extrinsic hallucinations as indicators, consistent with the findings of Hannigan et al. (6). The findings of this research contribute to the growing literature on diagnostic confidence, clinical reasoning, and awareness of practitioners in identifying hallucinated outputs during patient examination.

Extrinsic hallucination awareness demonstrated a significant positive impact on diagnosis confidence, indicating that health practitioners' knowledge of disease conditions and their ability to recognize hallucinated outputs enhance their diagnostic confidence (1, 48). This, in turn, enables practitioners to provide more adequate treatment to patients. Among the predictors of the external hallucination scale, awareness of data quality risks and awareness of documentation errors exhibited comparable outer loadings, indicating that both constructs load on extrinsic hallucination awareness.

Surprisingly, intrinsic hallucinations showed an insignificant impact on diagnostic confidence (H3). This finding contrasts with previous studies by Das et al. (27) and Fang et al. (28), which suggest that, despite the presence of biases, pattern overgeneralizations, and perceptual distortions, practitioners can easily identify hallucinated text due to their experience and awareness. This result further indicates that practitioners are conceptually able to recognize these outputs, as all the sub-constructs loaded significantly on the higher-order construct; however, they fail to perceive their real-time influence during diagnostic decision-making and prescription formulation. Hence, intrinsic hallucinations are unable to translate into diagnostic confidence. Further, from the perspective of epistemic risk theory, awareness of hallucinated outputs does not reduce risk; rather, it creates a deeper apprehension regarding the trustworthiness of Gen AI outputs. Therefore, unlike extrinsic hallucinations, intrinsic hallucinations involve deeper cognitive awareness. Hence, intrinsic hallucinations may function more as a trigger for epistemic awareness, which explicates their insignificant relationship with diagnostic confidence. These results further suggest that clinicians may place greater reliance on external indicators and observable patient symptoms, making extrinsic hallucination awareness exert a stronger impact on diagnostic confidence.

This research offers several implications for academicians and healthcare practitioners by providing insights into the responsible use of Gen AI. First, it conceptualizes “hallucinations” in the context of human diagnostics. This research, by validating intrinsic and extrinsic hallucinations, provides a unified lens for understanding perceptual distortions in healthcare professionals. The present study further integrates intrinsic and extrinsic hallucinations into a holistic framework of hallucination awareness, thereby providing a psychometric contribution to the literature. The validated HAS scale offers a novel instrument for assessing the awareness of hallucinated content among practitioners. The scale developed in this research may be used to design training modules and could be integrated into medical education programs to raise awareness of hallucinations and their impact on diagnosis and reasoning.

In addition, the study offers significant implications for improving clinical practice among healthcare professionals and across healthcare systems in general. Awareness of hallucinations enables healthcare practitioners to minimize the risk of misdiagnosis, promoting safer decision-making, especially in critical care settings. With adequate training, healthcare practitioners can more effectively integrate AI outputs with their experience and expertise. Such integration may support more accurate diagnoses while reducing over-reliance on technology. Considering that lapses in training data may propagate into model outcomes, improving data integrity and management is essential to reducing hallucinations. To address these challenges, curated datasets have been developed to meet the specific needs of specialized medical tasks. For instance, MedCPT (49) enhances access to scientific data, whereas MEDITRON-70B (44) is designed to improve therapeutic understanding. Both models are trained on carefully selected datasets tailored to their particular applications. Such domain-specific datasets enable LLMs to develop a deeper understanding of medical information, eventually improving accuracy and dependability. The findings further underscore the need for developing human–AI interaction guidelines that emphasize awareness of healthcare programs; such guidelines may help reduce algorithmic errors.

The findings provide a comprehensive framework for understanding how cognitive distortions influence clinical judgment. The instrument developed in this research serves as a practical tool for training, assessment, and clinical supervision, enabling healthcare organizations to strengthen perceptual discipline among practitioners. In conclusion, this research contributes to the existing body of knowledge by developing a scale on hallucinations and establishing its impact on diagnostic confidence.

## 7 Limitations and future scope

This research has a few limitations that could be considered by academicians working in the domain of Gen AI. As the field of Gen AI is dynamic, the severity of hallucinations may change over time, which could challenge the conceptualization of hallucinations as a stable construct. Hence, future researchers are encouraged to use diverse qualitative techniques, such as the expanded critical incidence technique (45,46,47), to further evaluate the universality of the developed scale. Snowball sampling was used in the qualitative phase to understand the usage of Gen AI, which may introduce bias. However, to mitigate this limitation, only participants with experience in using Gen AI were included. The study uses a cross-sectional design; a thorough understanding of the risks identified in this research may be investigated through longitudinal or experimental studies. Future research studies may validate other risks, such as prompting difficulties and quality dissatisfaction. Although this research provides evidence of initial reliability and validity, the absence of test–retest reliability assessment, measurement invariance testing, and cross-sample validation represents a limitation of this research, which could be taken up by future researchers. Future studies may replicate the measurement model across diverse healthcare groups and perform multigroup analyses. Another notable limitation of this study is the application of varimax rotation in the EFA, which may have constrained the estimation of correlations among factors. This could further distort the factorial solution, alter the loading pattern, and affect the interpretation. Future studies should employ oblique rotation methods that allow correlations among factors and provide a more robust representation of the relationships among constructs.

## Data Availability

The raw data supporting the conclusions of this article will be made available by the authors, without undue reservation.
