# Supplementary material for: Development and Evaluation of a Hallucination Awareness Scale for Healthcare Professionals and its impact on diagnostic confidence
Source: Front Digit Health. 2026 Mar 17;8:1772345. doi: 10.3389/fdgth.2026.1772345 (PMC13035798; doi:10.3389/fdgth.2026.1772345)
Supplement: Supplementary file 1 [file Datasheet1.pdf]

Supplementary Table 1: Scale Items

Construct	Scale Item (Items in italics are deleted items)
Awareness of Data Quality	I always verify whether the output by Gen AI is complete before using it for diagnosis.
	I can identify the inconsistencies of the clinical data.
	I can detect when the AI generated summaries do not match the actual record.
	I always verify the data sources whenever I feel that the information is unclear.
	I can make out when the content generated is outdated or irrelevant.
Awareness of Documentation Errors	I can easily identify the typological errors in the clinical output generated by AI.
	I can notice when automated documentation tools misinterpret clinical details.
	I can identify copy-paste errors in patient documentation.
	I can identify unexpected entries in electronic health records.
Awareness of Biases	I can recognize when AI is unable to justify clinical predictions of different genders.
	I can recognize when AI is unable to justify clinical predictions of different age groups
	I can find information that challenges my clinical impression
	I can identify false output due to faulty algorithm.
	Lack of bias detection systems discourage me to use Gen AI for routine purposes.
Awareness of Pattern overgeneralizations	I avoid using the output when similar symptoms have the same cause across genders.
	I always check whether interpretation by Gen AI is based on patterns rather than current evidence.
	I can notice when I generalize limited information.
Awareness of Perceptual Distortions	I recognize when reliance on Gen AI output may distract me from perceiving actual clinical signs.
	I am aware of cognitive fatigue when I accept the AI generated output without sufficient scrutiny.
	I can identify when Gen AI generated visual cues are misleading.
	I can identify ambiguous clinical signs and data generated by Gen AI.
Diagnostic Confidence	My awareness of possible Gen AI hallucination enhances my confidence while providing diagnostic recommendations.
	My alertness to hallucinations in Gen AI helps me to feel more confident in my clinical judgments.
	My ability to detect misleading or fabricated Gen AI content enhance my diagnostic confidence.

Supplementary Table 2: Descriptive Statistics

Name	Mean	Standard deviation	Excess kurtosis	Skewness
**I1**	4.029	0.894	1.004	-1.006
**I2**	4.096	0.832	1.106	-0.924
**I3**	4.029	0.811	0.505	-0.67
**I4**	3.936	0.993	0.858	-1.019
**I5**	3.27	1.223	-1.016	-0.242
**I6**	3.495	1.216	-0.79	-0.458
**I7**	3.576	1.303	-1.06	-0.491
**I8**	3.871	1.141	0.53	-1.116
**I9**	3.852	1.158	0.32	-1.056
**I10**	3.99	1.147	0.875	-1.267
**I11**	3.087	1.299	-1.275	0.033
**I12**	3.537	1.297	-1.033	-0.477
**I13**	3.849	1.091	0.276	-0.937
**I14**	3.678	1.125	0.134	-0.92
**I15**	3.961	0.948	0.57	-0.878
**I16**	4.154	0.779	2.756	-1.221
**I17**	4.129	0.792	1.036	-0.859
**I18**	4.296	0.723	2.577	-1.182
**I19**	4.36	0.67	2.353	-1.089
**I20**	4.045	1.167	0.699	-1.26
**I21**	4.039	1.181	0.477	-1.206
**I22**	4.267	0.783	1.083	-1.033
**I23**	4.347	0.732	2.908	-1.34
**I24**	4.299	0.702	0.667	-0.829
